# Bis(μ-methano­lato-κ^2^ *O*:*O*)bis­{[4-bromo-*N*′-(1-methyl-3-oxidobut-2-en-1-yl­idene-κ*O*)benzohydrazidato-κ^2^ *N*′,*O*]oxido­vanadium(V)}

**DOI:** 10.1107/S1600536811021647

**Published:** 2011-06-11

**Authors:** Hon Wee Wong, Kong Mun Lo, Seik Weng Ng

**Affiliations:** a Department of Chemistry, University of Malaya, 50603 Kuala Lumpur, Malaysia

## Abstract

The dinuclear compound, [V_2_(C_12_H_11_BrN_2_O_2_)_2_(CH_3_O)_2_O_2_], lies on a center of inversion. The doubly-deprotonated Schiff base *O*,*N*,*O*′-chelates to the V^V^ atom; two metal atoms are bridged by the methoxide units. The coordination geometry is a distorted octa­hedron. Weak inter­molecular C—H⋯N hydrogen bonding is present in the crystal structure. The bromo­phenyl unit is disordered over two positions, with the major component being in a 0.909 (6) proportion.

## Related literature

For the isotypic compound that has chlorine in place of bromine, see: Sarkar & Pal (2009 ▶).
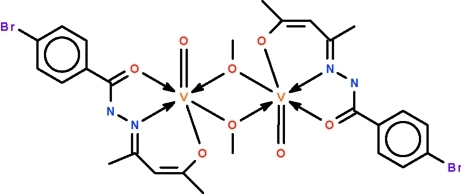


## Experimental

### 

#### Crystal data

[V_2_(C_12_H_11_BrN_2_O_2_)_2_(CH_3_O)_2_O_2_]
*M* *_r_* = 786.22
Monoclinic, 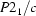

*a* = 8.7517 (5) Å
*b* = 12.3409 (7) Å
*c* = 13.9952 (8) Å
β = 101.8782 (7)°
*V* = 1479.17 (15) Å^3^
*Z* = 2
Mo *K*α radiation
μ = 3.39 mm^−1^
*T* = 100 K
0.30 × 0.25 × 0.20 mm

#### Data collection

Bruker SMART APEX diffractometer
Absorption correction: multi-scan (*SADABS*; Sheldrick, 1996 ▶) *T*_min_ = 0.430, *T*_max_ = 0.551
18572 measured reflections
3400 independent reflections
3106 reflections with *I* > 2σ(*I*)
*R*_int_ = 0.027

#### Refinement

*R*[*F* ^2^ > 2σ(*F* ^2^)] = 0.023
*wR*(*F* ^2^) = 0.060
*S* = 0.99
3400 reflections
221 parameters
44 restraints
H-atom parameters constrained
Δρ_max_ = 0.96 e Å^−3^
Δρ_min_ = −0.32 e Å^−3^

### 

Data collection: *APEX2* (Bruker, 2009 ▶); cell refinement: *SAINT* (Bruker, 2009 ▶); data reduction: *SAINT*; program(s) used to solve structure: *SHELXS97* (Sheldrick, 2008 ▶); program(s) used to refine structure: *SHELXL97* (Sheldrick, 2008 ▶); molecular graphics: *X-SEED* (Barbour, 2001 ▶); software used to prepare material for publication: *publCIF* (Westrip, 2010 ▶).

## Supplementary Material

Crystal structure: contains datablock(s) global, I. DOI: 10.1107/S1600536811021647/xu5231sup1.cif

Structure factors: contains datablock(s) I. DOI: 10.1107/S1600536811021647/xu5231Isup2.hkl

Additional supplementary materials:  crystallographic information; 3D view; checkCIF report

## Figures and Tables

**Table 1:** Selected bond lengths (Å)

V1—O1	1.9314 (13)
V1—O2	1.8607 (13)
V1—O3	1.5896 (14)
V1—O4	2.3459 (13)
V1—O4^i^	1.8289 (13)
V1—N2	2.0770 (15)

Symmetry code: (i) 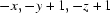.

**Table 2:** Hydrogen-bond geometry (Å, °)

*D*—H⋯*A*	*D*—H	H⋯*A*	*D*⋯*A*	*D*—H⋯*A*
C12—H12*C*⋯N1^ii^	0.96	2.59	3.541 (5)	169

Symmetry code: (ii) 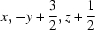.

**Fig. 1.**
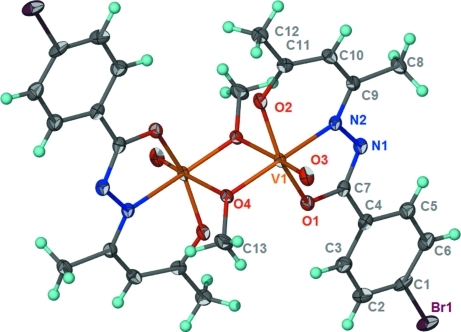
Thermal ellipsoid plot (Barbour, 2001) of [VO(C12H11BrN2O2)(CH3O)]2 at the 70% probability level; hydrogen atoms are drawn as spheres of arbitrary radius. The disorder in bromophenyl ring is not shown.

## supplementary crystallographic information

### Comment

A recent study detailed the crystal structure of [VO(C_12_H_11_ClN_2_O_2_)(CH_3_O)]_2_ (Sarkar & Pal, 2009). The title bromo analog (Scheme I) is isostructural, the two compounds crystallizing with matching cell dimensions. However, the title compound shows some disorder in the halophenyl portion (Fig. 1). Dinuclear [VO(C_12_H_11_BrN_2_O_2_)(CH_3_O)]_2_ lies on a center of inversion. The doubly-deprotonated Schiff base *O*,*N*,*O*'-chelates to the V^V^ atom; two metal centers are bridged by the methoxide unit. The geometry is an octahedron.

### Experimental

Bis(acetylacetonato)oxovanadium (0.67 g, 2.5 mmol) was heated with 4-bromobenzoic acid hydrazide (0.54 g, 2.5 mmol) in methanol (50 ml) for 1 h. The solution mixture was then filtered and upon slow cooling of the filtrate, dark brown crystals separated out.

### Refinement

Carbon-bound H-atoms were placed in calculated positions (C–H 0.95 to 0.98 Å) and were included in the refinement in the riding model approximation, with *U*(H) set to 1.2–1.5*U*(C).

The bromophenyl portion of the ligand is disordered over two positions. The pair of carbon–bromine distances were restrained to within ±0.01 Å each other. For the ring, the 1,2-related distances were restrained to 1.39±0.01 Å, the 1,3-related distances to 2.41±0.01 Å and the 1,4-related distances to 2.78±0.01 Å. Each seven-atom component was restrained to near planarity. Owing to the low degree of disordered, additional restraints were imposed on the primed bromine atom as well as the primed *para*-carbon atom. Distance restraints were applied so that the angle at the carbon atoms was approximately 120°. The isotropic temperature factors of the primed atoms were set to the anisotropic temperature factors of the unprimed ones. The disorder refined to 0.909 (1): 0.091.

### Crystal data

**Table:**

[V_2_(C_12_H_11_BrN_2_O_2_)_2_(CH_3_O)_2_O_2_]	*F*(000) = 784
*M**_r_* = 786.22	*D*_x_ = 1.765 Mg m^−^^3^
Monoclinic, *P*2_1_/*c*	Mo *K*α radiation, λ = 0.71073 Å
Hall symbol: -P 2ybc	Cell parameters from 8361 reflections
*a* = 8.7517 (5) Å	θ = 2.2–28.2°
*b* = 12.3409 (7) Å	µ = 3.39 mm^−^^1^
*c* = 13.9952 (8) Å	*T* = 100 K
β = 101.8782 (7)°	Prism, brown
*V* = 1479.17 (15) Å^3^	0.30 × 0.25 × 0.20 mm
*Z* = 2	

### Data collection

**Table:**

Bruker SMART APEX diffractometer	3400 independent reflections
Radiation source: fine-focus sealed tube	3106 reflections with *I* > 2σ(*I*)
graphite	*R*_int_ = 0.027
ω scans	θ_max_ = 27.5°, θ_min_ = 2.2°
Absorption correction: multi-scan (*SADABS*; Sheldrick, 1996)	*h* = −11→11
*T*_min_ = 0.430, *T*_max_ = 0.551	*k* = −16→16
18572 measured reflections	*l* = −18→18

### Refinement

**Table:**

Refinement on *F*^2^	Primary atom site location: structure-invariant direct methods
Least-squares matrix: full	Secondary atom site location: difference Fourier map
*R*[*F*^2^ > 2σ(*F*^2^)] = 0.023	Hydrogen site location: inferred from neighbouring sites
*wR*(*F*^2^) = 0.060	H-atom parameters constrained
*S* = 0.99	*w* = 1/[σ^2^(*F*_o_^2^) + (0.0308*P*)^2^ + 1.3638*P*] where *P* = (*F*_o_^2^ + 2*F*_c_^2^)/3
3400 reflections	(Δ/σ)_max_ = 0.001
221 parameters	Δρ_max_ = 0.96 e Å^−^^3^
44 restraints	Δρ_min_ = −0.32 e Å^−^^3^

### Fractional atomic coordinates and isotropic or equivalent isotropic displacement parameters (Å^2^)

**Table:**

	*x*	*y*	*z*	*U*_iso_*/*U*_eq_	Occ. (<1)
Br1	0.53762 (15)	0.29274 (14)	0.06235 (3)	0.0224 (2)	0.909 (6)
Br1'	0.5773 (11)	0.3313 (11)	0.0656 (3)	0.0164 (15)	0.091 (6)
V1	0.11414 (4)	0.60960 (2)	0.50419 (2)	0.01169 (8)	
O1	0.21661 (15)	0.52544 (10)	0.41937 (9)	0.0152 (3)	
O2	−0.04425 (16)	0.69868 (10)	0.52973 (9)	0.0149 (3)	
O3	0.25986 (16)	0.67280 (11)	0.56588 (10)	0.0179 (3)	
O4	−0.10415 (15)	0.51204 (10)	0.42270 (9)	0.0126 (2)	
N1	0.15083 (18)	0.65573 (13)	0.30152 (11)	0.0142 (3)	
N2	0.08999 (18)	0.70085 (12)	0.37726 (11)	0.0125 (3)	
C1	0.4474 (2)	0.3788 (2)	0.14888 (15)	0.0186 (5)	0.909 (6)
C1'	0.4680 (11)	0.4032 (11)	0.1483 (7)	0.019*	0.091 (6)
C2	0.4295 (3)	0.3360 (2)	0.23695 (18)	0.0202 (6)	0.909 (6)
H2	0.4659	0.2650	0.2558	0.024*	0.909 (6)
C2'	0.455 (3)	0.3560 (15)	0.2361 (13)	0.020*	0.091 (6)
H2'	0.5014	0.2875	0.2544	0.024*	0.091 (6)
C3	0.3573 (3)	0.39817 (19)	0.29774 (18)	0.0181 (5)	0.909 (6)
H3	0.3448	0.3697	0.3587	0.022*	0.909 (6)
C3'	0.373 (3)	0.4097 (14)	0.2974 (13)	0.018*	0.091 (6)
H3'	0.3632	0.3780	0.3577	0.022*	0.091 (6)
C4	0.3030 (2)	0.50212 (18)	0.26988 (16)	0.0154 (4)	0.909 (6)
C4'	0.3054 (10)	0.5095 (9)	0.2697 (8)	0.015*	0.091 (6)
C5	0.3265 (4)	0.5453 (2)	0.18142 (19)	0.0177 (4)	0.909 (6)
H5	0.2927	0.6168	0.1628	0.021*	0.909 (6)
C5'	0.319 (3)	0.5568 (15)	0.1819 (13)	0.018*	0.091 (6)
H5'	0.2728	0.6254	0.1635	0.021*	0.091 (6)
C6	0.3994 (3)	0.4833 (2)	0.12096 (18)	0.0194 (5)	0.909 (6)
H6	0.4161	0.5122	0.0610	0.023*	0.909 (6)
C6'	0.401 (3)	0.5032 (15)	0.1208 (13)	0.019*	0.091 (6)
H6'	0.4110	0.5349	0.0606	0.023*	0.091 (6)
C7	0.2196 (2)	0.56521 (15)	0.33309 (13)	0.0136 (3)	
C8	0.0615 (2)	0.86357 (16)	0.27592 (14)	0.0179 (4)	
H8A	0.1688	0.8559	0.2664	0.027*	
H8B	0.0386	0.9404	0.2839	0.027*	
H8C	−0.0112	0.8345	0.2189	0.027*	
C9	0.0435 (2)	0.80226 (14)	0.36548 (13)	0.0136 (3)	
C10	−0.0241 (2)	0.85486 (15)	0.43769 (13)	0.0149 (4)	
H10	−0.0407	0.9309	0.4319	0.018*	
C11	−0.0663 (2)	0.80336 (14)	0.51441 (13)	0.0138 (3)	
C12	−0.1443 (2)	0.85908 (15)	0.58647 (13)	0.0166 (4)	
H12A	−0.2375	0.8183	0.5932	0.025*	
H12B	−0.1745	0.9325	0.5635	0.025*	
H12C	−0.0718	0.8629	0.6499	0.025*	
C13	−0.2359 (2)	0.56198 (17)	0.36097 (15)	0.0209 (4)	
H13A	−0.2989	0.5065	0.3209	0.031*	
H13B	−0.1997	0.6151	0.3185	0.031*	
H13C	−0.2994	0.5986	0.4013	0.031*	

### Atomic displacement parameters (Å^2^)

**Table:**

	*U*^11^	*U*^22^	*U*^33^	*U*^12^	*U*^13^	*U*^23^
Br1	0.0193 (3)	0.0288 (5)	0.01969 (13)	0.0092 (3)	0.00564 (12)	−0.00508 (14)
Br1'	0.0125 (19)	0.018 (3)	0.0200 (12)	−0.001 (2)	0.0061 (10)	−0.0048 (12)
V1	0.01496 (16)	0.01018 (15)	0.01058 (15)	−0.00098 (11)	0.00417 (11)	0.00064 (11)
O1	0.0180 (6)	0.0146 (6)	0.0144 (6)	0.0015 (5)	0.0069 (5)	0.0018 (5)
O2	0.0203 (7)	0.0113 (6)	0.0148 (6)	−0.0004 (5)	0.0073 (5)	0.0003 (5)
O3	0.0210 (7)	0.0173 (7)	0.0152 (6)	−0.0046 (5)	0.0033 (5)	0.0012 (5)
O4	0.0145 (6)	0.0121 (6)	0.0114 (6)	0.0006 (5)	0.0030 (5)	0.0018 (5)
N1	0.0173 (8)	0.0144 (7)	0.0119 (7)	−0.0009 (6)	0.0056 (6)	−0.0015 (6)
N2	0.0140 (7)	0.0128 (7)	0.0114 (7)	−0.0009 (6)	0.0045 (6)	−0.0006 (6)
C1	0.0122 (9)	0.0263 (12)	0.0180 (10)	0.0034 (8)	0.0048 (8)	−0.0065 (9)
C2	0.0191 (13)	0.0208 (12)	0.0203 (10)	0.0059 (10)	0.0031 (9)	−0.0011 (9)
C3	0.0184 (12)	0.0198 (11)	0.0170 (10)	0.0021 (9)	0.0058 (8)	−0.0004 (8)
C4	0.0132 (9)	0.0176 (10)	0.0160 (9)	−0.0016 (8)	0.0046 (8)	−0.0020 (8)
C5	0.0184 (11)	0.0178 (11)	0.0180 (10)	0.0001 (9)	0.0061 (8)	−0.0006 (8)
C6	0.0174 (10)	0.0263 (13)	0.0160 (10)	0.0001 (10)	0.0067 (8)	−0.0012 (9)
C7	0.0131 (8)	0.0147 (8)	0.0135 (8)	−0.0036 (7)	0.0033 (7)	−0.0018 (7)
C8	0.0236 (10)	0.0159 (9)	0.0150 (9)	0.0020 (7)	0.0058 (7)	0.0037 (7)
C9	0.0126 (8)	0.0149 (9)	0.0130 (8)	−0.0012 (7)	0.0016 (7)	0.0010 (7)
C10	0.0189 (9)	0.0102 (8)	0.0154 (9)	0.0004 (7)	0.0033 (7)	−0.0002 (7)
C11	0.0141 (8)	0.0124 (8)	0.0142 (8)	0.0003 (6)	0.0011 (7)	−0.0023 (7)
C12	0.0204 (9)	0.0155 (9)	0.0146 (9)	0.0019 (7)	0.0053 (7)	−0.0020 (7)
C13	0.0184 (9)	0.0194 (10)	0.0218 (10)	0.0005 (7)	−0.0032 (8)	0.0065 (8)

### Geometric parameters (Å, °)

**Table:**

Br1—C1	1.9002 (19)	C3'—H3'	0.9500
Br1'—C1'	1.870 (8)	C4—C5	1.401 (3)
V1—O1	1.9314 (13)	C4—C7	1.479 (3)
V1—O2	1.8607 (13)	C4'—C5'	1.389 (9)
V1—O3	1.5896 (14)	C4'—C7	1.448 (8)
V1—O4	2.3459 (13)	C5—C6	1.389 (3)
V1—O4^i^	1.8289 (13)	C5—H5	0.9500
V1—N2	2.0770 (15)	C5'—C6'	1.390 (9)
O1—C7	1.309 (2)	C5'—H5'	0.9500
O2—C11	1.317 (2)	C6—H6	0.9500
O4—C13	1.430 (2)	C6'—H6'	0.9500
O4—V1^i^	1.8289 (13)	C8—C9	1.500 (2)
N1—C7	1.302 (2)	C8—H8A	0.9800
N1—N2	1.396 (2)	C8—H8B	0.9800
N2—C9	1.316 (2)	C8—H8C	0.9800
C1—C2	1.380 (3)	C9—C10	1.428 (3)
C1—C6	1.387 (3)	C10—C11	1.363 (3)
C1'—C6'	1.385 (9)	C10—H10	0.9500
C1'—C2'	1.386 (9)	C11—C12	1.496 (2)
C2—C3	1.391 (3)	C12—H12A	0.9800
C2—H2	0.9500	C12—H12B	0.9800
C2'—C3'	1.394 (9)	C12—H12C	0.9800
C2'—H2'	0.9500	C13—H13A	0.9800
C3—C4	1.395 (3)	C13—H13B	0.9800
C3—H3	0.9500	C13—H13C	0.9800
C3'—C4'	1.387 (9)		
			
O3—V1—O4^i^	102.96 (6)	C3'—C4'—C7	119.7 (7)
O3—V1—O2	98.74 (7)	C5'—C4'—C7	119.6 (7)
O4^i^—V1—O2	104.72 (6)	C6—C5—C4	119.90 (19)
O3—V1—O1	100.11 (7)	C6—C5—H5	120.1
O4^i^—V1—O1	89.10 (6)	C4—C5—H5	120.1
O2—V1—O1	153.42 (6)	C4'—C5'—C6'	119.5 (6)
O3—V1—N2	97.33 (6)	C4'—C5'—H5'	120.2
O4^i^—V1—N2	156.26 (6)	C6'—C5'—H5'	120.2
O2—V1—N2	83.99 (6)	C1—C6—C5	119.38 (19)
O1—V1—N2	75.18 (6)	C1—C6—H6	120.3
O3—V1—O4	176.30 (6)	C5—C6—H6	120.3
O4^i^—V1—O4	73.91 (6)	C1'—C6'—C5'	119.7 (6)
O2—V1—O4	80.35 (5)	C1'—C6'—H6'	120.1
O1—V1—O4	81.95 (5)	C5'—C6'—H6'	120.1
N2—V1—O4	86.17 (5)	N1—C7—O1	122.62 (16)
C7—O1—V1	117.75 (12)	N1—C7—C4'	117.5 (5)
C11—O2—V1	129.67 (12)	O1—C7—C4'	119.9 (5)
C13—O4—V1^i^	124.48 (12)	N1—C7—C4	119.95 (17)
C13—O4—V1	123.17 (11)	O1—C7—C4	117.43 (17)
V1^i^—O4—V1	106.09 (6)	C9—C8—H8A	109.5
C7—N1—N2	107.84 (14)	C9—C8—H8B	109.5
C9—N2—N1	116.14 (15)	H8A—C8—H8B	109.5
C9—N2—V1	126.68 (12)	C9—C8—H8C	109.5
N1—N2—V1	116.45 (11)	H8A—C8—H8C	109.5
C2—C1—C6	121.62 (19)	H8B—C8—H8C	109.5
C2—C1—Br1	119.58 (16)	N2—C9—C10	120.41 (16)
C6—C1—Br1	118.79 (16)	N2—C9—C8	120.12 (17)
C6'—C1'—C2'	120.9 (6)	C10—C9—C8	119.48 (16)
C6'—C1'—Br1'	119.5 (6)	C11—C10—C9	124.39 (17)
C2'—C1'—Br1'	119.7 (6)	C11—C10—H10	117.8
C1—C2—C3	118.99 (19)	C9—C10—H10	117.8
C1—C2—H2	120.5	O2—C11—C10	122.07 (17)
C3—C2—H2	120.5	O2—C11—C12	114.39 (16)
C1'—C2'—C3'	119.5 (6)	C10—C11—C12	123.52 (17)
C1'—C2'—H2'	120.3	C11—C12—H12A	109.5
C3'—C2'—H2'	120.3	C11—C12—H12B	109.5
C2—C3—C4	120.53 (19)	H12A—C12—H12B	109.5
C2—C3—H3	119.7	C11—C12—H12C	109.5
C4—C3—H3	119.7	H12A—C12—H12C	109.5
C4'—C3'—C2'	119.6 (6)	H12B—C12—H12C	109.5
C4'—C3'—H3'	120.2	O4—C13—H13A	109.5
C2'—C3'—H3'	120.2	O4—C13—H13B	109.5
C3—C4—C5	119.52 (18)	H13A—C13—H13B	109.5
C3—C4—C7	119.89 (19)	O4—C13—H13C	109.5
C5—C4—C7	120.59 (19)	H13A—C13—H13C	109.5
C3'—C4'—C5'	120.7 (6)	H13B—C13—H13C	109.5
			
O3—V1—O1—C7	−94.55 (13)	C2'—C3'—C4'—C7	179.7 (4)
O4^i^—V1—O1—C7	162.44 (13)	C3—C4—C5—C6	1.9 (3)
O2—V1—O1—C7	40.0 (2)	C7—C4—C5—C6	−177.37 (19)
N2—V1—O1—C7	0.43 (12)	C3'—C4'—C5'—C6'	0.2 (9)
O4—V1—O1—C7	88.57 (13)	C7—C4'—C5'—C6'	−179.8 (6)
O3—V1—O2—C11	56.92 (16)	C2—C1—C6—C5	−2.2 (3)
O4^i^—V1—O2—C11	162.88 (15)	Br1—C1—C6—C5	176.95 (15)
O1—V1—O2—C11	−77.8 (2)	C4—C5—C6—C1	0.3 (3)
N2—V1—O2—C11	−39.60 (16)	C2'—C1'—C6'—C5'	−0.2 (7)
O4—V1—O2—C11	−126.72 (16)	Br1'—C1'—C6'—C5'	179.7 (5)
O4^i^—V1—O4—C13	153.18 (16)	C4'—C5'—C6'—C1'	0.0 (9)
O2—V1—O4—C13	44.67 (14)	N2—N1—C7—O1	4.5 (2)
O1—V1—O4—C13	−115.43 (14)	N2—N1—C7—C4'	−173.8 (4)
N2—V1—O4—C13	−39.87 (14)	N2—N1—C7—C4	−176.61 (16)
O4^i^—V1—O4—V1^i^	0.0	V1—O1—C7—N1	−3.1 (2)
O2—V1—O4—V1^i^	−108.51 (7)	V1—O1—C7—C4'	175.2 (4)
O1—V1—O4—V1^i^	91.39 (6)	V1—O1—C7—C4	177.94 (13)
N2—V1—O4—V1^i^	166.95 (7)	C3'—C4'—C7—N1	−176.1 (15)
C7—N1—N2—C9	167.03 (16)	C5'—C4'—C7—N1	3.8 (16)
C7—N1—N2—V1	−3.84 (18)	C3'—C4'—C7—O1	5.5 (16)
O3—V1—N2—C9	−69.23 (16)	C5'—C4'—C7—O1	−174.6 (15)
O4^i^—V1—N2—C9	142.13 (16)	C3'—C4'—C7—C4	−40 (8)
O2—V1—N2—C9	28.85 (16)	C5'—C4'—C7—C4	140 (8)
O1—V1—N2—C9	−167.80 (16)	C3—C4—C7—N1	−170.83 (19)
O4—V1—N2—C9	109.53 (15)	C5—C4—C7—N1	8.4 (3)
O3—V1—N2—N1	100.55 (13)	C3—C4—C7—O1	8.1 (3)
O4^i^—V1—N2—N1	−48.1 (2)	C5—C4—C7—O1	−172.6 (2)
O2—V1—N2—N1	−161.38 (13)	C3—C4—C7—C4'	144 (8)
O1—V1—N2—N1	1.97 (11)	C5—C4—C7—C4'	−37 (8)
O4—V1—N2—N1	−80.70 (12)	N1—N2—C9—C10	178.40 (16)
C6—C1—C2—C3	1.91 (17)	V1—N2—C9—C10	−11.8 (3)
Br1—C1—C2—C3	−177.28 (11)	N1—N2—C9—C8	−1.7 (2)
C6'—C1'—C2'—C3'	0.2 (3)	V1—N2—C9—C8	168.13 (13)
Br1'—C1'—C2'—C3'	−179.8 (2)	N2—C9—C10—C11	−9.9 (3)
C1—C2—C3—C4	0.34 (15)	C8—C9—C10—C11	170.21 (18)
C1'—C2'—C3'—C4'	0.0 (3)	V1—O2—C11—C10	32.7 (3)
C2—C3—C4—C5	−2.2 (2)	V1—O2—C11—C12	−148.61 (13)
C2—C3—C4—C7	177.03 (14)	C9—C10—C11—O2	1.7 (3)
C2'—C3'—C4'—C5'	−0.2 (7)	C9—C10—C11—C12	−176.86 (18)

Symmetry codes: (i) −*x*, −*y*+1, −*z*+1.

### Hydrogen-bond geometry (Å, °)

**Table:**

*D*—H···*A*	*D*—H	H···*A*	*D*···*A*	*D*—H···*A*
C12—H12C···N1^ii^	0.96	2.59	3.541 (5)	169

Symmetry codes: (ii) *x*, −*y*+3/2, *z*+1/2.
